# Ag@SiO_2_ Core-shell Nanoparticles Embedded in a TiO_2_ Mesoporous Layer Substantially Improve the Performance of Perovskite Solar Cells

**DOI:** 10.3390/nano8090701

**Published:** 2018-09-08

**Authors:** Bao Wang, Xiangyu Zhu, Shuhan Li, Mengwei Chen, Haifei Lu, Yingping Yang

**Affiliations:** School of Science, Wuhan University of Technology, Wuhan 430070, China; 1174828021@whut.edu.cn (B.W.); zxy371653016@whut.edu.cn (X.Z.); 204410@whut.edu.cn (S.L.); mengwei.chen@whut.edu.cn (M.C.); haifeilv@whut.edu.cn (H.L.)

**Keywords:** Ag@SiO_2_ nanoparticles, perovskite solar cell, localized surface Plasmon resonance effect, scattering effect

## Abstract

In this study, Ag@SiO_2_ nanoparticles were synthesized by a modified Stöber method for preparing the TiO_2_ mesoporous layer of carbon counter electrode-based perovskite solar cells (PSCs) without a hole transporting layer. Compared with normal PSCs (without Ag@SiO_2_ incorporated in the TiO_2_ mesoporous layer), PSCs with an optimal content of Ag@SiO_2_ (0.3 wt. % Ag@SiO_2_-TiO_2_) show a 19.46% increase in their power conversion efficiency, from 12.23% to 14.61%, which is mainly attributed to the 13.89% enhancement of the short-circuit current density, from 20.23 mA/cm^2^ to 23.04 mA/cm^2^. These enhancements mainly contributed to the localized surface Plasmon resonance effect and the strong scattering effect of Ag@SiO_2_ nanoparticles. However, increasing the Ag@SiO_2_ concentration in the mesoporous layer past the optimum level cannot further increase the short-circuit current density and incident photon-to-electron conversion efficiency of the devices, which is primarily ascribed to the electron transport pathways being impeded by the insulating silica shells inside the TiO_2_ network.

## 1. Introduction

Over the past few years, much significant progress has been made in the development of perovskite solar cells (PSCs), such as the low-cost but high-efficiency CuSCN replacing spiro-OMeTAD [1], breakthroughs in large-area perovskite films fabrication [2] and constant improvements in power conversion efficiencies (PCEs), from an initial 3.9% to 23.2% [3,4]. Due to the excellent photovoltaic properties of perovskite, such as large light-harvesting coefficient, long carrier diffusion distance and high carrier mobility [5,6,7,8,9], PSCs have a bright prospect of partly replacing conventional energy. Recently, Bella et al. [10] made a 360-degree overview focusing on Cs-doping for PSCs to illustrate the excellent properties of Cs in perovskite-based devices. Bella et al. [11] explained the different electrochemical behavior of the amorphous and anatase phases of TiO2 and clarified the controversial mechanism of reversible Na storage in TiO_2_ nanotube arrays. Zhang et al. [12] prepared TiO_2_@C aerogel as superior anode for lithium-ion batteries which exhibited a superior high rate and stable performance. Bella et al. [13] introduced the photoanodes and cathodes exclusively based on polymeric materials into dye-sensitized solar cells (DSSCs) and obtained a 5.33% power conversion efficiency. Galliano et al. [14] made a multivariate study on aqueous DSSCs to provide a new approach of finding the optimal operating conditions of any new energy device with solid mathematical and statistical bases. Abate et al. [15] analyzed the critical points about currently limiting the industrial production of PSCs.

However, being high efficient light harvesters, the thickness of organo-metal halide perovskites should be limited, reducing the lead content as much as possible, due to environmental and health concerns [16,17]. Therefore, the light capture capability of perovskite absorbers (PA) must be appropriately regulated to reach optimum efficiency. One strategy to improve the utilization rate of sunlight of PA could be incorporating metal nanoparticles (NPs) into PSCs.

When the photon frequency matches the natural oscillation frequency of the metal NP surface electrons, the conduction band (CB) electrons of noble metal NPs are induced to the coherent surface Plasmon oscillations [18], which is termed localized surface Plasmon resonance (LSPR). Consequently, this causes a huge increase in light absorption and scattering [19,20]. LSPR is macroscopically expressed as a strong characteristic absorption peak in the ultraviolet-visible (UV-vis) absorption spectrum. Mixing metal NPs into PSCs could not only enhance the optical properties related to size, shape, structure and environment [21] but also make use of electrical characteristics which are reported to help dissociate excitons and transport carriers more efficiently [22]. 

Certainly, there are numerous academic papers on the incorporation of metal nanomaterials into photovoltaic devices for better performance via their optical and electrical properties [23,24,25]. For example, Wang et al. [26] significantly improved the PCEs by adding 70 nm-Au NPs into polymer bulk heterojunction solar cells. Kulkarni et al. [27] utilized photoinduced absorption spectroscopy to improve the generation of charge carrier by using silver nanoprisms in organic photovoltaic devices. Batmunkh et al. [28] introduced the plasmonic Au nanostars into PSCs for enhancing photon absorption and thus improving device performance. Furthermore, an insulating SiO_2_ shell was added onto the surface of metal NPs to inhibit corrosion [29] and exciton quenching [30], however, this did not influence the LSPR effect. Metal-oxide core-shell NPs have widespread application in PSCs [31,32,33,34,35,36], as well as in DSSCs [37] and organic solar cells [38,39]. However, there has been little research on incorporating Ag@SiO_2_ NPs into low-cost free-hole-conductor PSCs with carbon counter electrodes. Ag NPs show superior scattering efficiency compared to any other metal NPs [40].

In this work, we adopted a modified Stöber [41] method to prepare Ag@SiO_2_ NPs for manufacturing the TiO_2_ mesoporous layer of PSCs. Through ultrasound treatment and continuous stirring, Ag@SiO_2_ NPs and mesoporous TiO_2_ pulp suspension were thoroughly homogenized. PSCs with Ag@SiO_2_ NPs exhibited better performance, evidenced by their UV-vis absorption spectra, *J*-*V* curves and incident photon-to-electron conversion efficiency (IPCE) spectra, because of the strong LSPR and scattering effects. With the hole-conductor-free and carbon-based counter electrode, the PSC devices gained a 19.46% enhancement of PCE, from 12.23% to 14.61% and a 13.89% increase in short-circuit current density (*J*_SC_), from 20.23 mA/cm^2^ to 23.04 mA/cm^2^.

## 2. Results and Discussion

**Figure 1:**
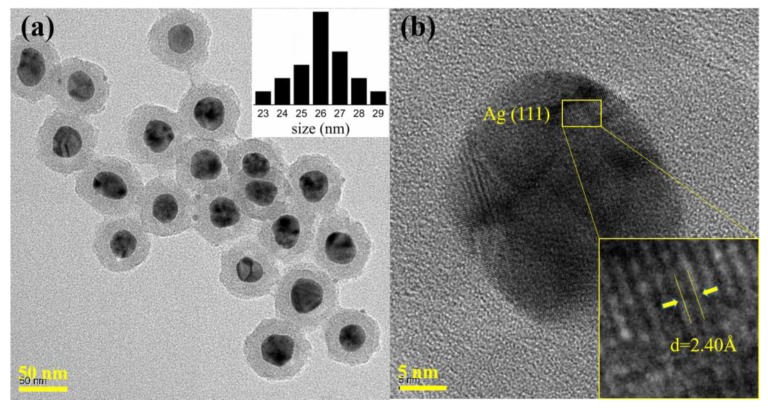
(**a**) TEM image and size distribution results of Ag@SiO_2_ NPs; (**b**) HRTEM image of a Ag@SiO_2_ NP.

We successfully synthesized Ag NPs with a polyol method, whereas the silica shells coated on the Ag NPs were deposited uniformly with a modified Stöber method. The obtained NPs were then characterized by field emission transmission electron microscopy (FE-TEM, JEOL Ldt., Tokyo, Japan), high-resolution TEM (HRTEM, JEOL Ldt., Tokyo, Japan) and UV-vis absorption spectra (UV3600, Shimadzu Corporation, Tokyo, Japan). As depicted in Figure 1a, Ag NPs were evenly dispersed in ethanol, with the size distribution results in the upper right corner of the image. The nucleation sizes of the Ag NPs were almost equal and basically concentrated at 26 nm, while the thickness of the silica shells averaged 14 nm. Figure 1b shows the HRTEM image of a Ag@SiO_2_ NP. The lattice fringes of the Ag NP can be observed and were measured at about 2.40 Å matched with the (111) crystallographic planes of Ag NPs, which is consistent with the available literature [42].

**Figure 2:**
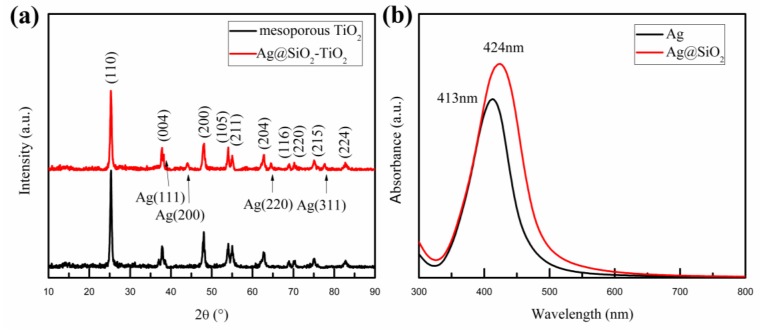
(**a**) XRD patterns of mesoporous TiO_2_ and the Ag@SiO_2_-TiO_2_; (**b**) Optical absorption spectra of 26 nm-Ag NPs and Ag@SiO_2_ NPs dispersed in alcohol.

Figure 2a shows the X-ray diffraction (XRD) patterns of mesoporous TiO_2_ and the mesoporous TiO_2_ mixed with Ag@SiO_2_ NPs. A series of matching diffraction peaks characteristic for TiO_2_ were observed at 25°, 38°, 48°, 54°, 55°, 63°, 69°, 70°, 75°, 83°, which correspond to the anatase phase according to the standard PDF card (JCPDS Card No. 89-4921). These characteristic diffraction peaks are respectively attributed to the (101), (004), (200), (105), (211), (204), (116), (220), (215), (224) crystal planes of anatase phase TiO_2_. Simultaneously, there are several additional diffraction peaks in Figure 2a at the angles of 38°, 44°, 65° and 78°; these correspond to the reflections of the (111), (200), (220) and (311) crystal planes of the Ag NPs [43]. Furthermore, no other characteristic diffraction peaks of Ag oxide and SiO_2_ were observed, which implies that Ag was present in crystal form and SiO_2_ in an amorphous state in the prepared samples. Figure 2b exhibits the optical absorption spectra of 26 nm-Ag NPs and Ag@SiO_2_ NPs dispersed in alcohol solution. Compared to Ag NPs, the Plasmon resonance peak of Ag@SiO_2_ NPs had an obvious red-shift of around 10 nm, which was caused by the large refractive index of the silica shell [44,45]. The red-shifted distances of Ag@SiO_2_ NPs are related to the thickness of the silica shells, which are relative to the added amount of ethylsilicate [46]. The dielectric SiO_2_ shells coated around Ag NPs could prevent the recombination of carriers on the surface of metal NPs [30], even further enhancing the LSPR effect [47], as already reported.

**Figure 3:**
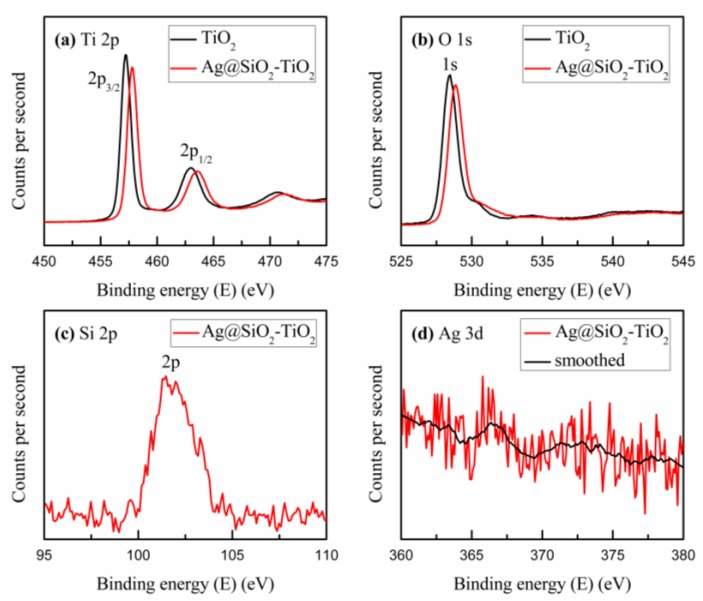
XPS survey spectra acquired from mesoporous TiO_2_ and the Ag@SiO_2_-TiO_2_: (**a**) Ti 2p; (**b**) O 1s; (**c**) Si 2p; (**d**) Ag 3d.

To confirm the physical state of chemical elements in the mesoporous TiO_2_ mixed with Ag@SiO_2_ NPs, X-ray photoelectron spectroscopy (XPS) was performed to assess the binding energy of chemical elements in the sample. Figure 3 shows the Ti, O, Si, Ag binding states and the surface composition of the NPs. As pictured in Figure 3a,b, the binding energies of 457.2 eV, 463.0 eV and 528.9 eV, correspond to the peaks of Ti 2p_3/2_, Ti 2p_1/2_ and O 1s respectively. However, compared with the pristine TiO_2_, the binding energies of the Ti 2p_3/2_, Ti 2p_1/2_ and O 1s electrons increased to 0.6 eV, 0.6 eV and 0.4 eV, respectively, after the addition of Ag@SiO_2_ NPs, which implies that a few electrons of the Ti and O atoms were trapped by Ag@SiO_2_ NPs due to the local electromagnetic field [48]. The decrease in electrons around Ti and O atoms implies a reduction in electron cloud densities around the Ti and O atoms and thus an increase in the binding energies. Figure 3c shows the peak of Si 2p at the binding energy of 103 eV, which is in accord with the standard database. In Figure 3d, no distinct peak can be found, which suggests that the 10 nm silica shells around the Ag NPs are sufficiently thick to attenuate the Ag photoelectrons generated by the X-ray [49]. Nonetheless, after a simple smoothing treatment, a weak peak was observed at the binding energy of 367 eV, corresponding to the peak of Ag 3d_5/2_. Compared with the standard binding energy of Ag^0^ at 368.25 eV, there is a small decrease that may be attributed to the few Ag NPs not covered by the silica shells. While calcinating the samples, the increased temperature led to the growth of the Ag NPs, which decreased the Ag 3d_5/2_ electronic binding energy [48]. Both the XRD patterns and XPS survey spectra indicate that the Ag@SiO_2_ NPs were successfully mixed into the TiO_2_ mesoporous layer.

**Figure 4:**
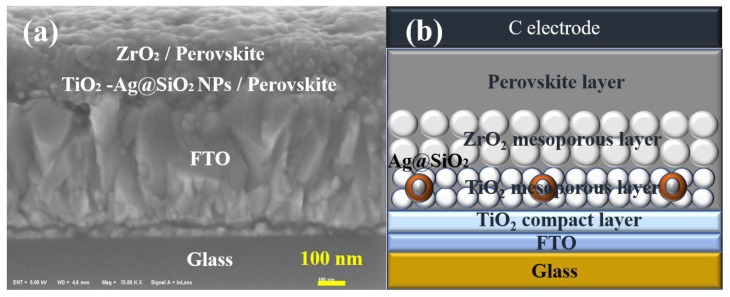
(**a**) Scanning Electron Microscope (SEM) image of a cross section of a complete PSC mixed with Ag@SiO_2_ NPs; (**b**) Structural representation of a whole PSC device mixed with Ag@SiO_2_ NPs.

As depicted in Figure 4, a whole PSC device includes glass, FTO, a TiO_2_ compact layer, TiO_2_ mesoporous layer, ZrO_2_ mesoporous layer, perovskite layer and carbon counter electrode from the bottom up. According to the scale plate in Figure 4a, the thickness of each layer of the solar cell could be diagrammatically calculated. The thickness of the FTO, TiO_2_ compact layer, TiO_2_ mesoporous layer/perovskite and ZrO_2_ mesoporous layer/perovskite was roughly 500 nm, 30 nm 100 nm and 100 nm, respectively. In addition, the carbon counter electrode film was almost 30 μm, which is upon the perovskite film by screen printing. In the FTO/c-TiO_2_/m-TiO_2_/m-ZrO_2_/perovskite/carbon architecture-based PCSs, the ZrO_2_ layer acting as a scaffold to host the perovskite absorber with TiO_2_ layer, can block the flow of photogenerated electrons to the back contact and prevent recombination with the holes from the perovskite at the back contact [50].

**Figure 5:**
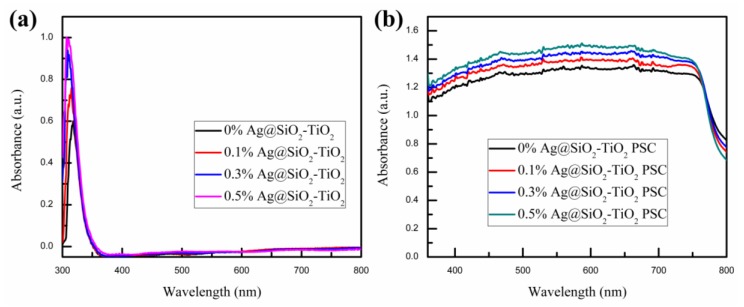
UV-vis absorption spectra of (**a**) pristine TiO_2_ and TiO_2_ mixed with Ag@SiO_2_ NPs mesoporous film samples and (**b**) PSCs with pristine TiO_2_ and TiO_2_ mixed with Ag@SiO_2_ NPs mesoporous film.

The UV-vis absorption spectra of pristine TiO_2_ and TiO_2_ mixed with Ag@SiO_2_ NPs mesoporous film samples are presented in Figure 5a. The black absorption curve represents the absorption intensity of pristine mesoporous TiO_2_ in the wavelength of 300–800 nm, which has an absorption peak at approximately 325 nm and is nearly zero in 360–800 nm, as previously reported by Yue et al. [51]. Compared with the other absorption curves, it is evident that the intensity of the absorption peak at around 325 nm became stronger as the content of Ag@SiO_2_ NPs in the mesoporous TiO_2_ layer gradually increased. As pictured in Figure 5b, the UV-vis absorption spectra of PSCs with pristine TiO_2_ and TiO_2_ mixed with Ag@SiO_2_ NPs followed the same trend in Figure 5a. The enhancement could be attributed to the LSPR effect and scattering effect of Ag@SiO_2_ NPs. By absorbing photons in the wavelength of the plasmonic resonance band, Ag@SiO_2_ NPs produced an intense LSPR effect which stimulated a strong electromagnetic field [52]. The intense local electromagnetic field could cause bandgap excitation for the nearby TiO_2_ NPs, enhancing their photocatalytic abilities, which could generate more electron-hole pairs in the TiO_2_ NPs [53]. The scattering effect of Ag@SiO_2_ NPs is mainly due to the scattering of incident light by the Ag NPs, which increases the length of the light propagation path and then further improves light trapping and the light utilization rate [34]. Macroscopically, the LSPR and scattering effects of Ag@SiO_2_ NPs are shown in the absorption spectrum, as depicted in Figure 5. As the content of Ag@SiO_2_ NPs increases, the LSPR effect and scattering effect increase gradually, which correlates with the absorption curves. According to previous research, the LSPR effect and scattering effect are dependent on the size of the metal NPs, with the LSPR effect decreasing as the metal NPs grow and the scattering effect increasing as the metal NPs grow [21,40]. Based on Zhang’s calculations, for Ag NPs, the LSPR effect reaches the maximum absorption efficiency at 20 nm, while the scattering effect achieves maximum scattering efficiency at 40 nm [54]. For the relatively small 26 nm-Ag NPs, the LSPR effect plays a more significant role than the scattering effect in the PSC devices [40,54].

**Figure 6:**
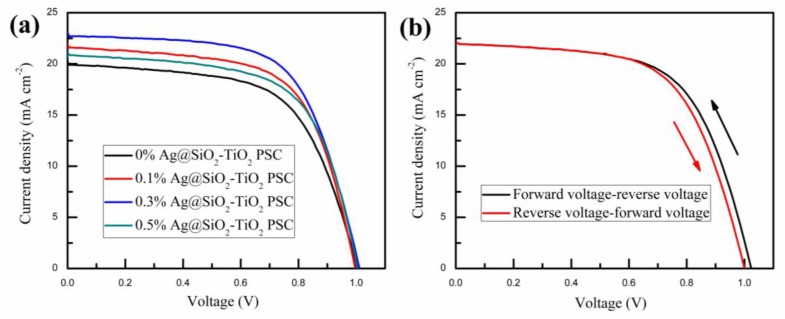
(**a**) *J*-*V* curves of PSC devices based on mesoporous TiO_2_ film mixed with different amounts of Ag@SiO_2_ NPs; (**b**) *J*-*V* curves scanned form forward voltage to reverse voltage and scanned form reverse voltage to forward voltage.

**Figure 7:**
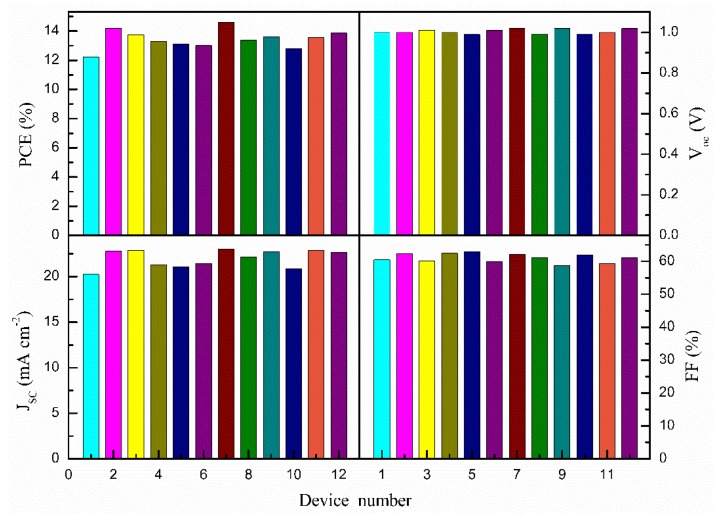
Histograms of photovoltaic parameters for 12 devices (the same batch of samples) based on mesoporous TiO_2_ film mixed with 0.3 wt. % Ag@SiO_2_ NPs.

The PSCs based on mesoporous TiO_2_ film mixed with different amounts of Ag@SiO_2_ NPs were measured via scanning from −1.1 V to short circuit at a scan rate of 150 mV/s under AM 1.5G irradiation (100 mW/cm^2^) in ambient air to characterize their photovoltaic performance. Except for the process of spin-coating the perovskite solution, which was accomplished inside a nitrogen glovebox, all other processes, including spin-coating the precursor solution of the compact TiO_2_ layer, spin-coating the pulp suspension of mesoporous TiO_2_ mixed with different amounts of Ag@SiO_2_ NPs and mesoporous ZrO_2_ and screen-printing the carbon counter electrode, were performed in ambient air, at room temperature. As shown in Figure 6a, the PSC devices containing 0.3 wt. % Ag@SiO_2_ NPs had the most outstanding photovoltaic performance. Summarized photovoltaic parameters of PSCs based on mesoporous TiO_2_ film mixed with different amounts of Ag@SiO_2_ NPs are described in Table 1, which corresponds to Figure 6a. Figure 7 shows the histograms of photovoltaic parameters for 12 devices (the same batch of samples) based on mesoporous TiO_2_ film mixed with 0.3 wt. % Ag@SiO_2_ NPs. Compared with normal PSCs (with no plasmonic NPs), the photovoltaic performance of cells containing 0.3 wt. % Ag@SiO_2_ NPs was enhanced 19.46% regarding their PCE (from 12.23% to 14.61%) and 13.89% at *J*_SC_, from 20.23 mA/cm^2^ to 23.04 mA/cm^2^, while the open-circuit voltages (*V*_OC_) and fill factor (FF) of the PSCs rose and fell slightly at 1.00 V and 61.00%, respectively. The enhancements could be attributed to the incorporation of Ag@SiO_2_ NPs, which generated strong LSPR and scattering effects, improving the light trapping and utilization rate. The *V*_OC_ of the PSC devices are related to the CBs of TiO_2_ and perovskite [55] and were not influenced by the Ag@SiO_2_ NPs. As described in Figure 6a and Table 1, as the number of Ag@SiO_2_ NPs increased gradually, the *J_SC_* and PCE initially increased, reaching the optimal value with the addition of 0.3 wt. % Ag@SiO_2_ NPs and then decreased. The enhancement was due to the strong increase in LSPR effect and scattering effect, while the diminution may be attributed to the offset between plasmonic light trapping effects and the decrease in electron transmission routes with the increased Ag@SiO_2_ loading [56]. Certainly, PSCs usually suffer from a hysteresis effect in *J*-*V* measurements, which is related with measurement settings and device properties [57,58,59]. In the FTO/c-TiO_2_/m-TiO_2_/m-ZrO_2_/perovskite/carbon architecture-based PCSs, the properties of the c-TiO_2_ layer and corresponding interfaces significantly affect the *J*-*V* hysteresis [60]. As depicted in Figure 6b and Table 2, the photocurrent, voltage, efficiency and fill factor for the forward-reverse scan were a little higher than those for the reverse-forward scan, which indicated that the hysteresis effect was a little influence in this architecture.

**Figure 8:**
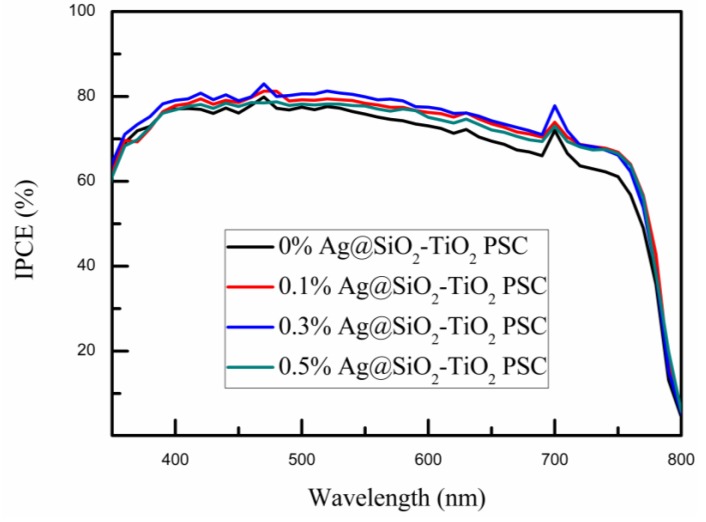
IPCE spectra of PSC devices based on mesoporous TiO_2_ film mixed with different amounts of Ag@SiO_2_ NPs.

The IPCE spectra of PSC devices were measured to further demonstrate the impact of Ag@SiO_2_ NPs on the photoelectric properties of PSCs. It is evident that the trends shown in Figure 8 were consistent with the *J*-*V* curves depicted in Figure 6. With increased loading of Ag@SiO_2_ NPs, the IPCE reached its maximum at the concentration of 0.3 wt. %, followed by a decrease as the Ag@SiO_2_ NPs content increased past the optimal value. As pictured in Figure 8, in the wavelength of 500–750 nm, substantial increases were observed compared to the reference device (the black curve). This may be attributed to the perovskite material, which can efficiently use light in the short wavelength but behaves relatively poor in the long wavelength region of 600–800 nm [61,62]. Therefore, the LSPR effect and scattering effect of Ag@SiO_2_ NPs performed relatively better in the long wavelength. According to previous research, it is crucial to obtain the electron transport paths simple, straight and with few crystal boundaries to collect more electrons [63,64]. However, with increased concentration of Ag@SiO_2_ NPs, electrons became impeded by the insulating silica shells on the way to the electrode inside the TiO_2_ network [56]. Hence, as the Ag@SiO_2_ concentration increased past the optimal value, the *J_SC_* and IPCE curves presented a downward trend.

**Figure 9:**
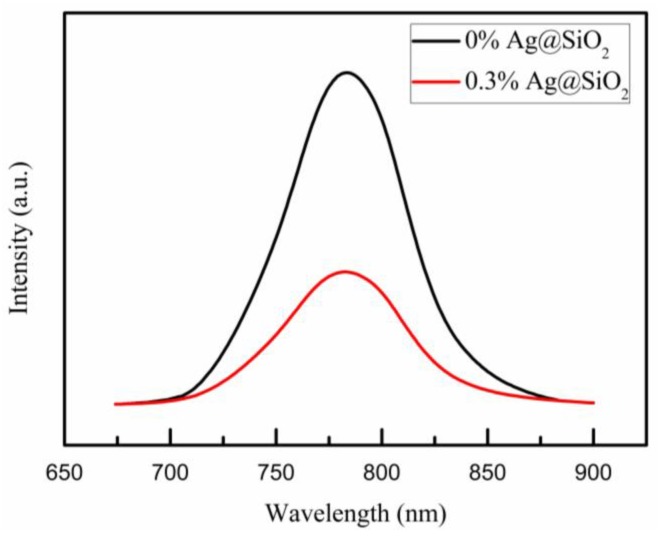
PL spectra of compact TiO_2_/Ag@SiO_2_-TiO_2_/ZrO_2_/perovskite films at room temperature of 296 K.

**Figure 10:**
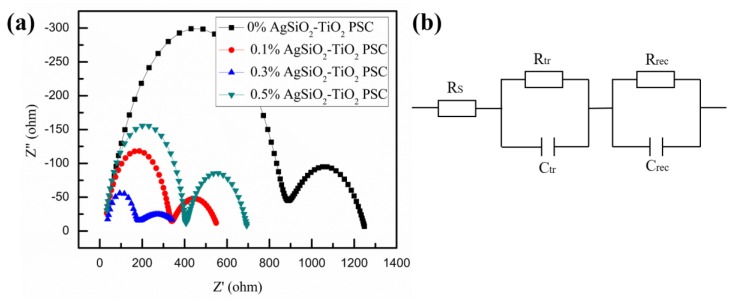
(**a**) Nyquist curves of the PSC devices based on different amounts of Ag@SiO_2_ NPs films and (**b**) the equivalent circuit employed to fit the plots.

To understand the charge transport properties of the devices in more details, we studied the photoluminescence (PL) at room temperature of 296 K. In Figure 9, it was obvious that there was a clear quenching of the steady-state PL for the compact TiO_2_/Ag@SiO_2_-TiO_2_/ZrO_2_/perovskite films compared with the films without Ag@SiO_2_ NPs, which was contributed from ionization of the excitons and enhanced charge separation [65]. The electrochemical impedance spectroscopy (EIS) analyses were performed for frequencies of 10 mHz to 10 MHz at a bias of 0.8 V under simulated AM 1.5G radiation (irradiance of 100 mW/cm^2^) to further understand charge transport and charge recombination in the devices. As described in Figure 10a, there were two semicircles in the Nyquist plots, which corresponded to the high-frequency region and low-frequency region, respectively. The left semicircle represented charge transfer and charge recombination at the perovskite/C electrode interface associated with the charge transfer resistance (*R*_tr_), while the right one represented charge transfer and charge recombination at the perovskite/TiO_2_ interface related to the recombination resistance (*R*_rec_) [66,67]. The series resistance (*R*_S_) value is inversely related to FF value [68]. In Table 3, the R_S_ of 0% Ag@SiO_2_-TiO_2_ PSC was slightly larger than the others, which was in keeping with the FF values in Table 1 basically. For the recombination resistance *R*_rec_ at the perovskite/TiO_2_ interface, it was clear that with the Ag@SiO_2_ NPs mixed into the films *R*_rec_ values decreased immediately, which indicated that there were more electrons generated duo to the LSPR effect and scattering effect of Ag@SiO_2_ NPs and sequentially more electrons were recombined at the perovskite/TiO_2_ interface. To analyze the charge transfer at the perovskite/C electrode interface, the *R*_tr_ values were shown in Table 3, which decreased as the content of Ag@SiO_2_ NPs decreased gradually and increased when the concentration was higher than the optimal value. The results of *R*_tr_ indicated that there were more holes transported to the C electrode and with increased concentration of Ag@SiO_2_ NPs higher than the optimal value, holes were impeded by the insulating silica shells on the way to the C electrode inside the TiO_2_/ZrO_2_ network. Therefore, 0.3% Ag@SiO_2_-TiO_2_ PSC got the best photovoltaic properties in this work, in keeping with *J-V* curves and IPCE spectra.

## 3. Materials and Methods 

### 3.1. Synthesis of Ag NPs

The Ag NPs were prepared *via* a polyol method, according to previously reported methods with slight modifications [40,53,69,70]. First, 3 g polyvinylpyrrolidone (PVP) was dissolved in 40 mL ethylene glycol and heated up to 120 °C slowly, in an oil bath, with continuous stirring, until it dissolved completely. Subsequently, 0.5 g silver nitrate (AgNO_3_) were dissolved in 20 mL ethylene glycol with vigorous stirring. After the AgNO_3_ was fully dissolved, the mixture was added to the ethylene glycol solution of PVP dropwise and the resulting solution was kept at 120 °C, for 1 h, under stirring. After the colloidal solution cooled down to room temperature, moderate deionized water was added to the solution, followed by centrifugation. Finally, the precipitate was washed with deionized water and acetone for several times, then dried at 40 °C to obtain the Ag NPs.

### 3.2. Synthesis of Ag@SiO_2_ NPs

The Ag@SiO_2_ NPs were synthesized by a modified Stöber method [40,53,71]. Firstly, 120 mg Ag NPs were uniformly dispersed in 120 mL alcohol with vigorous stirring. Secondly, after 20 min of ultrasonication, 2.5 mL ammonium hydroxide, 2.5 mL deionized water and 0.3 mL ethylsilicate were added to the solution dropwise. Finally, the solution was stirred for 12 h at room temperature and Ag@SiO_2_ NPs were obtained by centrifugation, wash (ethyl alcohol for several times) and desiccation.

### 3.3. Device Fabrication

First, to prepare the Ag@SiO_2_-TiO_2_ with different ratios (0.1–0.5 wt. % ), 4 g alcohol were added to 1 g TiO_2_ sizing agent (solid content: 20%) followed by stirring for 6 h. Then, 0.2–1 mg Ag@SiO_2_ NPs powder were added to the mixture followed by stirring for at least 48 h. Normally, Ag@SiO_2_ NPs disperse in a mixture more uniformly after 30 min of ultrasonication. Second, fluorine-doped tin oxide-patterned glass substrates (7 Ω/sq) were cleaned sequentially with detergent, acetone, isopropanol and ethanol, each followed by 30 min of ultrasonication. Third, the TiO_2_ compact layer was deposited by spin coating 35 μL TiO_2_ precursor solution (1 mL titanium disopropoxide bis added into 19 mL ethanol) at 4000 r/min for 25 s and annealed at 150 °C for 10 min. Then, 35 μL TiO_2_ precursor solution was spin-coated on the substrate again at 4000 r/min for 25 s and annealed at 150 °C for 10 min, followed by annealing at 500 °C for 30 min. Fourth, after cooling down to room temperature, the TiO_2_ mesoporous layer was deposited on the top of the TiO_2_ compact layer by spin coating appropriate TiO_2_ sizing agent mixed with Ag@SiO_2_ NPs at 3500 r/min for 25 s, after which the glass substrates were heated at 150 °C for 10 min and annealed at 500 °C for 30 min. Fifth, the ZrO_2_ paste was spun onto the substrates at 5000 r/min for 25 s, then the glass substrates were heated at 150 °C for 10 min and sintered at 500 °C for 30 min. Lastly, the (CH_3_NH_3_)PbI_3_ perovskite film was fabricated by spin coating 35 μL perovskite precursor solution at 1000 r/min for 10 s and 4000 r/min for 20 s, followed by annealing at 100 °C for 5–10 min. The perovskite precursor solution was prepared by dissolving 462 mg PbI_2_ and 178 mg (CH_3_NH_3_)I in 600 mg dimethylformamide (DMF) and 78 mg dimethyl sulfoxide (DMSO). During 5 s at 4000 r/min, about 200 μL methylbenzene were dropped on the spinning substrates to ensure the fast crystallization of perovskite by extracting the solvent of the perovskite precursor solution.

### 3.4. Characterization Techniques

FE-TEM images were photographed by a JEM-2100F (JEOL Ldt., Tokyo, Japan). XRD patterns were acquired from an X-ray diffractometer (Advance D8, AXS, Rigaku Corporation, Tokyo, Japan). XPS (ESCALAB 250Xi, Thermo Fisher Scientific, Waltham, Massachusetts, USA) was employed to analyze the chemical elements of the samples. SEM (JSM-IT300, JEOL Ldt., Tokyo, Japan) was used to scan the surface and section of the as-prepared samples. The light-absorption spectra were obtained *via* UV-vis spectrophotometry (UV3600, Shimadzu Corporation, Tokyo, Japan). The photocurrent-voltage (*J*-*V*) characteristics were acquired from an electrochemical workstation (Zahner Company, Kronach, Germany) with a solar light simulator (Oriel Sol3A, Newport Corporation, Irvine, CA, USA), under simulated AM 1.5G illumination, at 100 mW/cm^2^ intensity. Finally, IPCE (Newport Corporation, Irvine, CA, USA) was employed to study the quantum efficiency of the PSCs. The PL spectra were measured with a fluorescence spectrometer (RF-6000, Shimadzu Corporation, Tokyo, Japan). The EIS analyses were conducted on an electrochemical workstation (Zahner Company, Kronach, Germany) for frequencies of 10 mHz to 10 MHz at a bias of 0.8 V under simulated AM 1.5G radiation (irradiance of 100 mW/cm^2^) with an alternating current (AC) signal amplitude of 10 mV at room temperature.

## 4. Conclusions

In this study, we successfully synthesized Ag@SiO_2_ NPs with a modified Stöber method and developed PSCs with different contents of Ag@SiO_2_ NPs. TEM images, XRD patterns and XPS survey spectra showed that Ag was present in nanocrystal form and that Ag@SiO_2_ NPs were thoroughly mixed into the TiO_2_ mesoporous layer. UV-vis absorption spectra demonstrated that the optical absorption capacity of mesoporous TiO_2_ films and PSC devices can be improved with increased loading of Ag@SiO_2_ NPs (0–0.5 wt. %), which could be attributed to the strong LSPR and scattering effects. Finally, the variation of *J*-*V*, IPCE and EIS between devices with different Ag@SiO_2_ concentrations was assessed and the results indicated that the electron transporting paths were blocked by the Ag@SiO_2_ NPs when their concentration was higher than the optimal value (0.3 wt. %). Compared to the normal PSCs (0 wt. % Ag@SiO_2_-TiO_2_), the PSCs incorporating the optimal content of NPs exhibited a 19.46% improvement of the PCE (from 12.23% to 14.61%) and 13.89% improvement of *J_SC_* (from 20.23 mA/cm^2^ to 23.04 mA/cm^2^). Our method of using Ag@SiO_2_ NPs provides a new approach to boosting highly efficient hole-conductor-free PSCs based on carbon counter electrodes.

## Figures and Tables

**Table 1:** Summarized photovoltaic parameters of PSC devices based on mesoporous TiO_2_ film mixed with different amounts of Ag@SiO_2_ NPs.

Samples	*V*_OC_ (V)	*J*_SC_ (mA/cm^2^)	PCE (%)	FF (%)
0% Ag@SiO_2_-TiO_2_	1.00	20.23	12.23	60.45
0.1% Ag@SiO_2_-TiO_2_	0.99	21.90	13.65	62.96
0.3% Ag@SiO_2_-TiO_2_	1.02	23.04	14.61	62.17
0.5% Ag@SiO_2_-TiO_2_	0.99	21.16	13.20	63.01

**Table 2:** Summarized photovoltaic parameters of PSC devices scanned form forward voltage to reverse voltage and scanned form reverse voltage to forward voltage.

Samples	*V*_OC_ (V)	*J*_SC_ (mA/cm^2^)	PCE (%)	FF (%)
Forward-reverse	1.02	22.23	13.88	61.21
Reverse- forward	1.00	22.22	13.50	60.76

**Table 3:** Summary of EIS parameters of PSCs based on different amounts of Ag@SiO_2_ NPs films.

Samples	*R*_S_ (Ω)	*R*_tr_ (Ω)	*R*_rec_ (Ω)
0% Ag@SiO_2_-TiO_2_ PSC	29.75	842.4	381.6
0.1% Ag@SiO_2_-TiO_2_ PSC	22.77	308.2	231.1
0.3% Ag@SiO_2_-TiO_2_ PSC	25.02	125.8	243.0
0.5% Ag@SiO_2_-TiO_2_ PSC	24.84	379.0	294.1
